# The Michael J. Fox Foundation for Parkinson’s Research Strategy to Advance Therapeutic Development of PINK1 and Parkin

**DOI:** 10.3390/biom9080296

**Published:** 2019-07-24

**Authors:** Shalini Padmanabhan, Nicole K. Polinski, Liliana B. Menalled, Marco A.S. Baptista, Brian K. Fiske

**Affiliations:** The Michael J. Fox Foundation for Parkinson’s Research, Grand Central Station, P.O. Box 4777, New York, NY 10120, USA

**Keywords:** Parkinson’s disease, genetic, Parkin, PINK1, mitochondria, biomarkers, therapeutic development, mitophagy, antibodies

## Abstract

The role of mitochondria in Parkinson’s disease (PD) has been investigated since the 1980s and is gaining attention with recent advances in PD genetics research. Mutations in *PRKN* and PTEN-Induced Putative Kinase 1 (*PINK1*) are well-established causes of autosomal recessive early-onset PD. Genetic and biochemical studies have revealed that PINK1 and Parkin proteins function together in the same biological pathway to govern mitochondrial quality control. These proteins have also been implicated in the regulation of innate and adaptive immunity and other mitochondrial functions. Additionally, structural studies on Parkin have delineated an activation mechanism and have identified druggable regions that are currently being explored by academic and industry groups. To de-risk therapeutic development for these genetic targets, The Michael J. Fox Foundation for Parkinson’s Research (MJFF) has deployed a strategic funding and enabling framework that brings together the research community to discuss important breakthroughs and challenges in research on PINK1-Parkin biology, supports collaborative initiatives to further our understanding within this field and develops high-quality research tools and assays that are widely available to all researchers. The Foundation’s efforts are leading to significant advances in understanding of the underlying biology of these genes, proteins and pathways and in the development of Parkinson’s therapies.

## 1. Background

Parkinson’s disease (PD) is a chronic, progressive neurodegenerative disorder characterized by loss of dopaminergic neurons in the substantia nigra (SN) leading to motor dysfunction but can also impact other pathways implicated in non-motor disease manifestations [1]. The etiology of PD remains largely unknown; however, genetic studies over the past two decades point to crucial roles for certain genes as contributors to disease onset and progression. Although these genetic differences are only associated with a small proportion of PD cases, they can reveal the importance of certain cellular pathways and processes that may be disrupted in PD more broadly. Translating these insights into new diagnostics or therapeutics for PD patients remains a challenge and requires significant additional research to (i) identify the functional role of the genes and mutations under normal and disease conditions, (ii) determine if the protein or another component of the pathway can be modulated, (iii) elucidate the structure of the proteins to identify druggable regions and (iv) confirm that modulating the protein is safe in preclinical models. The Michael J. Fox Foundation for Parkinson’s Research (MJFF) deploys strategic funding to fill the knowledge gaps and also acts as a convener of experts; bringing together academic and industry partners to discuss challenges hindering therapeutic development, fostering collaborations using novel approaches to address these challenges and promoting data and resource-sharing between teams. Most importantly, the MJFF plays a proactive role to develop and characterize essential preclinical tools and animal models to ensure that high-quality resources are widely available and research findings are robust and reproducible.

Translation of understanding of the PD-associated gene leucine-rich repeat kinase 2 (LRRK2) into therapeutic development provides an excellent example of how a framework for strategic investment and coordinated action can accelerate progress. First genetically linked to PD in 2004 [2,3], LRRK2 protein has been shown to be present largely in the cytoplasm but also associates with the mitochondrial outer membrane [4,5]. Early studies demonstrated that mutations in *LRRK2* increase its encoded protein’s kinase activity [6] providing strong rationale for development of LRRK2 kinase inhibitors for the treatment of PD. However, a lack of protein structural insight, limited understanding of the upstream and downstream cellular signaling networks for LRRK2, including its endogenous protein substrates, and uncertainty around the broader role of LRRK2 in idiopathic forms of PD were clear obstacles along the path to clinical development of LRRK2-targeted therapies. Moreover, preclinical studies in genetic knockouts or with LRRK2 inhibitors suggested impact of reduced LRRK2 activity on tissues within the lung and kidney, highlighting potential issues for safety when moving these therapies into human testing. Applying focused strategic funding and collaboration, MJFF worked closely with community stakeholders to address these and other important questions. Today, MJFF-funded researchers have identified endogenous protein substrates for LRRK2 [7], built improved models of the functional structure of the LRRK2 protein [8,9], clarified understanding of the impact of inhibiting LRRK2 kinase activity in preclinical models [10] and demonstrated that increased kinase activity is found in the idiopathic PD (iPD) population [11,12]. These and other crucial data have increased confidence for continued clinical development of LRRK2-targeted therapies, the first of which are now in Phase 1 clinical trials [NCT03710707 and NCT03976349].

Using a similar framework, we are working with the community stakeholders to explore the potential for developing treatments targeting two additional proteins with genetic links to PD: Parkin and PTEN-induced putative kinase protein 1 (PINK1). Below, we briefly describe the rationale for targeting these proteins and our perspective on the biological, therapeutic, biomarker and resource needs critical for successful translation of these proteins into therapies for people with PD.

## 2. Mitochondrial Dysfunction in PD

A growing body of evidence points to mitochondrial dysfunction as a contributor to PD pathogenesis. Mitochondria are traditionally viewed as cellular powerhouses. However, the broader role of mitochondria involves a host of functions important for maintaining neuronal health and survival. Early evidence of mitochondrial involvement in PD came from serendipitous identification of several so-called “frozen addicts” in the 1970s [13]. These individuals had injected heroin that had been laced with 1-methyl-4-phenyl-1,2,3,6-tetrahydropyridine (MPTP) and began exhibiting PD-like motor symptoms, due to the selective potency of this toxin to dopamine neurons. Since MPP^+^, the major metabolite of MPTP, inhibits complex 1 of the electron transport chain [14,15], these case reports provided strong evidence for mitochondrial dysfunction as a possible cause of parkinsonism. Moreover, use of MPTP is now a powerful tool for modeling the disease in preclinical laboratory models of PD [16]. Subsequent studies in patient tissues reported deficits in mitochondrial complex 1 activity [15,17,18] and accumulation of mitochondrial DNA (mtDNA) mutations [19].

In 1997, mutations in the *SNCA* gene were associated with familial PD. Over the next decade, several additional genes such as *PRKN*, *PINK1* and *LRRK2*, amongst others, were identified and their functional roles were extensively characterized in various genetic and toxin PD models. These studies demonstrated the important role of these genes in modulating mitochondrial bioenergetics, dynamics and quality control [20]. Interestingly, these functions appear to be a convergence point for both familial and iPD, thus highlighting mitochondrial dysfunction as a common denominator of PD pathogenesis [21].

## 3. PINK1 and Parkin PD

Mutations in *PRKN* and *PINK1* can explain approximately 13% of autosomal recessive PD with disease onset under 45 years of age [22]. Heterozygous variants in these genes are also found at a higher rate in the PD population compared to healthy controls suggesting a role in increasing genetic risk for PD [23,24,25,26]. Typically, autosomal recessive PD linked to *PRKN* and *PINK1* mutations has an earlier disease onset, slower progression and a remarkable levodopa response [27]. Pathologically, these forms of PD are characterized by cell loss in the SN with conflicting evidence on the presence of Lewy bodies [28].

**Figure 1:**
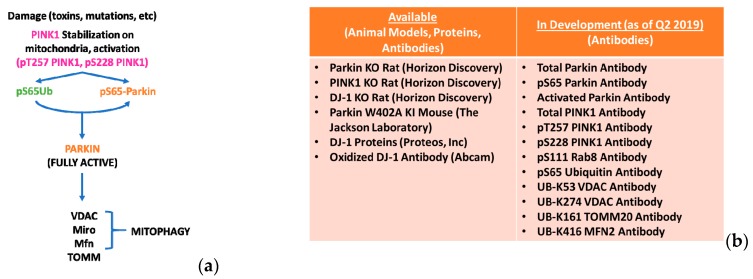
PTEN-Induced Putative Kinase 1 (PINK1) and Parkin pathway preclinical tools and animal models (**a**) The multiple players within the PINK1-Parkin-mitophagy pathway are indicated. Upon mitochondrial damage, PINK1 is stabilized on the mitochondrial membrane and gets activated. Once active, PINK1 phosphorylates ubiquitin that in turn binds to Parkin and causes Parkin translocation to the mitochondria. PINK1 also phosphorylates Parkin in the mitochondria that results in a conformational change in Parkin and renders it fully active. Parkin then ubiquitinates itself and several other mitochondrial proteins, the most prominent of which are listed in the figure. This labels the damaged mitochondria for degradation by the lysosome (mitophagy); (**b**) The various tools generated and distributed through the Michael J. Fox Foundation (MJFF) are listed. For detailed information on the status of their availability and distribution partner, please visit www.michaeljfox.org/toolscatalog.

Linkage of the *PRKN* and *PINK1* genes to Parkinson’s disease led to some seminal papers in the field of mitochondrial biology in PD. In 2006, studies in drosophila demonstrated that loss of either *PRKN* or *PINK1* led to degeneration of the flight muscles and dopamine neurodegeneration [29]. This work also demonstrated that both PINK1 and Parkin were part of a linear molecular pathway where Parkin was downstream of PINK1 [30,31]. The currently accepted model (Figure 1a) suggests that in response to mitochondrial depolarization, PINK1 is stabilized at the mitochondria and activates Parkin. Once active, Parkin ubiquitinates several mitochondrial proteins and tags the mitochondria for degradation by the lysosome, a process termed “mitophagy.” PD-associated mutations in *PINK1* and *PRKN* dampen this process, [32] resulting in the accumulation of damaged mitochondria and cell death. Conversely, enhancing the expression of these proteins is neuroprotective in various PD preclinical models [33,34,35]. Building on understanding of the functional effects of these proteins in in vitro and in vivo models, four independent groups crystallized the Parkin protein [36,37,38,39] and outlined a detailed multi-step activation process [40,41]. These structural and biochemical studies provide insights on the normal and pathological role for Parkin. More importantly, these studies provide knowledge on regions of the protein that could be leveraged for therapeutic development.

The strong genetic link to PD coupled with knowledge of the crystal structure and evidence of the neuroprotective potential of activating these proteins provided a strong rationale for MJFF to recognize these targets as priorities. In 2013, the Foundation developed an initiative to translate PINK1-Parkin understanding into meaningful therapies for people with Parkinson’s disease. Through this initiative, our goals are to bring together researchers working on these targets to discuss the state of the field for identification of key challenges, and strategies for addressing these challenges to expedite therapeutic development of these genetic targets. In this article, we will provide a perspective on our understanding of the field, including the key challenges and ongoing efforts to fill translational gaps across four major goals within the Foundation’s PINK1-Parkin portfolio:

- Increase understanding of the biological function of PINK1 and Parkin in PD
- Facilitate the development of PINK1-Parkin pathway-targeting therapies
- Develop, optimize and validate biomarker assays to assess PINK1-Parkin pathway activation
- Develop, characterize and distribute preclinical tools and animal models to facilitate PD research

Additional information on completed and ongoing studies can be found on www.michaeljfox.org/funded-studies.

### 3.1. Increase Understanding of the Biological Function of PINK1 and Parkin in PD

Compared to other PD-related genes and the proteins they encode, the functions of PINK1 and Parkin proteins have been well-elucidated. Unlike other proteins implicated in the pathogenesis of PD, the crystal structure for inhibited Parkin is known and studies have extensively characterized the role of these proteins in mitophagy in various cellular models. The biggest bottlenecks that prevent the translation of these biological findings into PD therapies are (i) the lack of detailed knowledge on the structure of the normal and mutated versions of the proteins, (ii) the reliance on harsh stressors to activate this pathway, and (iii) limited understanding of the exact downstream molecular players involved in PINK1-Parkin-mediated neuroprotection.

The initial crystal structures of closed Parkin provided the first model to understand domain-domain interactions to maintain Parkin in an inhibited state [36,37,38,39]. However, we have learned only recently how inhibition is relieved by domain reorganization as structures of intermediate activated conformations of Parkin were revealed [40,42]. These studies have identified additional regions that could be targeted within the protein to activate Parkin, enabling further development of Parkin activators. On the other hand, it has been challenging to express and purify the human PINK1 protein for crystal structure studies and only insect PINK1 structures are currently available [42,43,44]. Since these studies have been a major driver for therapeutic programs, MJFF hosted a workshop in 2019 that brought together structural biologists and drug development experts from industry to discuss scientific and technical challenges that delay these efforts. The outcomes of these discussions have resulted in various groups working together to share tools, platforms and protocols to continue efforts focused on validating the Parkin structure using different crystallography techniques and developing new approaches to express and purify the human PINK1 protein.

There is consensus that activation of the PINK1-Parkin pathway promotes mitophagy. However, these proteins are usually over-expressed in cellular models and mitophagy can only be measured upon activation of this pathway by mitochondrial depolarizing agents, therefore providing an artificial system that may not truly reflect mechanisms of its endogenous regulation. In an attempt to determine if mitophagy can be monitored in vivo in preclinical models, at endogenous levels of these proteins, a new mitophagy reporter mouse model was recently generated [45]. Surprisingly, deleting *PINK1* or *PRKN* failed to influence basal mitophagy in the SN, suggesting the involvement of other proteins in this process. MJFF is currently supporting efforts to determine if PD-relevant stressors such as toxins, alpha-synuclein or mutations in the mitochondrial DNA polymerase result in observable differences in mitophagy in the knockout (KO) models. This study will provide additional insights on the role for Parkin in stress-induced mitophagy and identify other mechanisms that activate the pathway. In parallel, we are also working with groups to identify novel upstream modifiers of PINK1-Parkin-mediated mitophagy using a mass spectrometry-based approach [46]. Through the use of a genome-wide screen, we aim to uncover new targets, that impact the various steps within this pathway, for therapeutic development and increase our understanding of the mechanisms of PINK1-Parkin activation.

In addition to investigating the role of PINK1 and Parkin proteins in mitophagy and PD pathogenesis, another area of active MJFF support is the identification of additional functional roles of these proteins and the mechanisms by which they impart neuroprotection. MJFF-funded efforts are informing the field of new roles for these proteins in mitochondrial biogenesis [47,48,49], innate immunity [50,51], and adaptive immunity [52]. There are also reports indicating a role for the ubiquitin-proteasome system [53] and mitochondrial lipids [54] in mediating the effects of PINK1 and Parkin proteins on mitochondrial quality control and mitochondrial complex-1 deficits. As these studies utilized different models and focused solely on one pathway, future efforts to assess the relative contribution of each of these pathways in response to a variety of PD-relevant stressors will be important for continuing this line of research.

### 3.2. Facilitate the Development of PINK1-Parkin Pathway-Targeting Therapies

**Figure 2:**
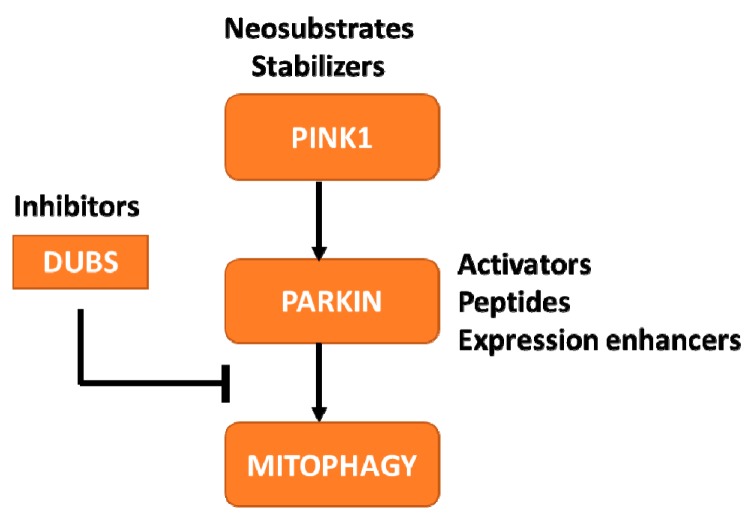
Therapeutic approaches to increase PINK1-Parkin mediated mitophagy. The schematic indicates the proteins within the pathway that are currently being targeted for developing PD therapies, along with the therapeutic approach being pursued.

In an effort to develop better treatments and ultimately a cure for PD, MJFF helps groups with promising therapies to advance their compounds to the clinic. Our role in this process can range from that of a funder to a partner/collaborator advising on models, biomarkers and connecting groups with experts in the field to problem-solve and de-risk therapeutic development for these targets. Several companies have initiated therapeutic programs targeting the PINK1-Parkin pathway. Observations from preclinical studies have demonstrated that enhancing expression or activity of these proteins or inhibiting the de-ubiquitinating enzymes (DUBs) can have therapeutic benefit [55]. For each of these targets, diverse approaches are currently being explored by academic labs and industry groups (Figure 2).

Knowledge of the crystal structures of these proteins has proven beneficial for designing activator compounds. For example, the Parkin protein contains several domain-domain interactions that maintain the protein in the inhibited state, allowing for specific targeting of these regions to promote opening and activation of the protein [40,41]. The structural knowledge has also permitted the use of in silico artificial intelligence- based screening technologies to nominate potential lead molecules. Another example of crystal structures promoting drug development within this pathway can be found in the DUBs—proteins that promote removal of ubiquitin chains to restrain pathway activation. Of the 100+ human DUBs, there is evidence supporting the role of USP8 [56,57], USP15 [58] and USP30 [59,60] in the modulation of Parkin auto-ubiquitination and Parkin-mediated mitochondrial ubiquitination. Of the three, USP30 is being pursued for PD therapeutic development based on studies demonstrating the ability of USP30 KO to enhance mitophagy in cellular systems and rescue PD-like motor phenotypes in *PINK1* and *PRKN* KO drosophila [59,60]. These efforts have been furthered through the available crystal structure for USP30 which has enabled companies to generate selective, USP30 inhibitors [61]. The Foundation supports promising and novel approaches to target these proteins through funding initiatives and by connecting industry partners with protein crystallography experts to further elucidate the mechanism by which compounds interact with proteins to alter activity and confer neuroprotection.

Apart from small molecule activators, the field is pursuing alternate strategies for modulating these proteins. For Parkin, peptide delivery to restore functional proteins is being investigated based on reports from preclinical studies indicating that mere overexpression could be beneficial. These alternate strategies are especially important for the protein PINK1 as the crystal structure of the human protein remains to be elucidated. PINK1 is present at low levels under basal conditions due to cleavage by the mitochondrial intramembrane protease, PARL, and subsequent proteasomal degradation. However, upon PARL inhibition, PINK1 is stabilized at the mitochondria where the kinase can be activated by autophosphorylation [62] to boost Parkin-dependent mitophagy [63]. The Foundation is supporting two novel approaches to increase PINK1 activity-inhibition of PARL to stabilize PINK1 and amplification of the catalytic activity of PINK1 using a neo-substrate approach [64]. As many of these therapeutic programs are in early stages, it is unclear which of these approaches will yield compounds that can cross the blood-brain barrier and prove efficacious in preclinical models. However, a promising trend has been observed in the increase in investment in therapies targeting PINK1, Parkin, and DUB proteins, as well as other targets within this pathway that may likely progress to therapeutic programs in the near future [65,66,67,68].

As companies advance with their lead candidates, a common challenge they face is in identifying the best animal model to test therapeutic efficacy. The Foundation has funded several groups in the past to characterize the *PINK1* and *PRKN* KO animal models and is currently funding additional characterization of these models and new models such as the *PINK1*/*PRKN* double KO rats. Once characterized, these models will provide the field with additional data on robust and reliable endpoints that could be monitored after treatment with PINK1-Parkin therapies. For instance, a recent report demonstrated significant alterations in cytokine levels in blood after stressing *PINK1* and *PRKN* KO mice through an exhaustive exercise paradigm [51]. MJFF has initiated a collaboration to establish this model at a contract research organization and validate these important findings. Importantly, the establishment of this model at a contract research organization will provide companies working in this area with access to a reproducible model system to test their therapies.

Finally, a successful drug discovery program includes the assessments of safety liability of modulating the target. PINK1 and Parkin proteins are highly expressed in many peripheral tissues including skeletal muscle and heart, emphasizing the importance of demonstrating that activation of this pathway does not pose any safety concerns. In order to address these concerns at a broader field-wide level, MJFF is promoting the sharing of tool compounds across industry groups and academia. This strategy was employed by MJFF previously for investigating the safety of LRRK2 kinase inhibitors. In a large-scale, multi-industry group collaboration led by MJFF, three companies provided LRRK2 inhibitor tool compounds to assess on-target safety liability. This collaborative effort demonstrated that histopathological changes observed in the lung after treatment were reversible and did not result in any changes in pulmonary functional assessments [10]. Such studies are highly informative and benefit the entire field, but are lacking for PINK1 and Parkin due to the unavailability of potent and selective tool compounds.

In an attempt to de-risk therapeutic development for targets within the PINK1-Parkin pathway, MJFF is encouraging the sharing of tool compounds across groups. As one example of this, MJFF is collaborating with An2H Discovery to test their Parkin activator tool compounds in a safety study in rats. Although this is an important step in advancing compounds that mimic the An2H Parkin activators, it does not address safety risks for other compounds targeting different regions of the protein or other targets within the pathway. While the Foundation is eager to initiate a safety study in collaboration with various partners with active programs in PINK1, Parkin or DUBs as was done in the LRRK2 field, the lack of advanced programs and tool compounds has presented a challenge. To circumvent this issue, the Foundation has organized a collaborative study with four academic groups to identify activating mutations in *PRKN* that would increase its ligase activity without destabilizing the protein. By characterizing the various mutations and generating knockin mouse models harboring these mutations, MJFF hopes to provide suitable models to address potential safety liabilities induced by sustained Parkin activation. With these de-risking strategies, MJFF hopes to encourage investments in developing new therapies for the iPD patient population.

### 3.3. Develop, Optimize and Validate Biomarker Assays to Assess PINK1-Parkin Pathway Activation

A successful clinical trial program for PINK1 and Parkin therapeutics is dependent on access to biomarker assays to assist in patient selection/stratification and interpretation of the biologic impact of therapy. The PINK1-Parkin driven mitophagy and inflammation pathways are the strongest indicators of target modulation that are showing promise for further testing in biomarker studies. As therapeutic programs for PINK1 and Parkin are targeted for the iPD population, efforts to determine if these pathways are deregulated in human samples from *PINK1* and *PRKN* mutation carriers need to be extended to iPD subjects to identify a subset of the iPD population with similar mitochondrial phenotypes. Efforts to build this link between PINK1/Parkin-PD and iPD have traditionally focused on the use of fibroblasts or induced pluripotent stem cells (iPSCs) from *PINK1* and *PRKN* mutation carriers and iPD cases to study the effects of these proteins on mitophagy in endogenous, human model systems [69,70,71]. Although these studies are providing interesting insights on the role of these proteins in triggering mitophagy and have resulted in the identification of other modifiers of this pathway, use of these cells for biomarker studies is unfeasible due to the low-throughput nature. MJFF is currently working with groups to determine if outcomes from these fibroblast and iPSC studies could be expanded into other easily accessible human matrices such as blood and immune cells.

Furthermore, MJFF is supporting antibody-based assays that could be used for analyzing different readouts of the PINK1-Parkin pathway in patient biofluids. Two such studies include immunoassay development for a downstream Parkin substrate, Miro [70] and ubiquitin phosphorylated at serine 65 (pS65 Ubiquitin) [72]. Prior studies assessing levels of these proteins in fibroblasts and postmortem brain tissues have indicated altered pathway regulation in *PINK1* and *PRKN* mutation carriers as well as iPD patients. These studies are providing us with hints on biological mechanisms that are shared between the brain and periphery, and if successful, would provide utility for assessing pathway regulation in human peripheral cells and biofluids. Although several other pathway proteins could serve as excellent markers of mitophagy, progress in the biomarker field for these proteins has been slow as high-quality antibodies to detect these proteins and their activated versions are lacking. While MJFF generates these tools to aid in biomarker efforts, we also work with investigators who have access to antibodies and assays to determine if these could be distributed and shared with the community.

The recent research indicating dysregulated PINK1 and Parkin pathways may alter the NLRP3 inflammasome [50] and the cytosolic DNA sensor- cGAS/STING [51] pathways has provided additional opportunities for biomarker assay development and testing. These studies demonstrated that PINK1 and Parkin protein activation can increase production of NLRP3-mediated IL1 and IL-18 as well as increase cGAS/STING-mediated IL-6 levels in *PRKN* and/or *PINK1* patient-derived macrophages and serum, respectively. Unfortunately, the analysis of these cytokines was limited to a small number of subjects since access to human samples from *PINK1* and *PRKN* mutation carriers is limited given the rare nature of these mutations. To address the need for samples from *PINK1* and *PRKN* mutation carriers, MJFF worked with partners to conduct a systematic online survey to identify PD mutation carriers across several global clinical sites. Through this survey MJFF has identified over a thousand patients with mutations in *PINK1* and *PRKN* genes [73] and is continuing to collect demographic, clinical and genetic data along with information on biosample availability. The goal is to make this information available to the research community to facilitate collaborations between groups with assay expertise and clinical sites to enable the identification, optimization and validation of PINK1-Parkin pathway biomarkers.

**Figure 3:**
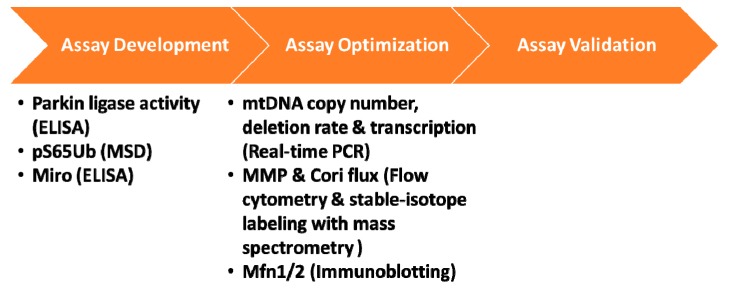
PINK1-Parkin pathway biomarkers. The schematic demonstrates the three essential phases for a biomarker assay: development, optimization and validation. Most biomarker efforts for PINK1 and Parkin fall under the assay development and assay optimization phases. MJFF-funded studies are listed along with the platform used for analysis in parenthesis. The assay development efforts use recombinant proteins while assay optimization efforts are in PBMCs derived from *PINK1* and/or *PRKN* mutation cases and controls.

Various aspects of mitochondrial function could be affected in PD and while studies actively seek to develop and validate the right biomarker assays for assessing PINK1-Parkin pathway in iPD, the field is also leveraging existing mitochondrial biomarker assays that are capable of detecting altered mitochondrial function, beyond mitophagy, in *PINK1* and *PRKN* mutation carriers and iPD cases. In 2017 MJFF launched a funding initiative soliciting proposals from groups with expertise in mitochondrial biomarker assays. By expanding our network in this open grant callout, MJFF identified many promising research programs and funded several groups to assess a variety of readouts of mitochondrial function, including mtDNA copy number, transcription, mitochondrial membrane potential and Cori Cycle flux in peripheral blood-derived mononuclear cells from mutation carriers and controls (Figure 3). Outcomes of these studies will help determine if these assessments could be used to segregate the iPD population based on their mitochondrial phenotype and may provide additional endpoints for future clinical trials.

### 3.4. Develop, Characterize and Distribute Preclinical Tools and Animal Models to Facilitate PD Research

Researchers rely on critical tools, including molecular tools and pre-clinical models, to understand the cause and mechanisms underlying Parkinson’s disease and to develop effective treatments. Unfortunately, such tools are often lacking, inaccessible or not properly quality controlled. To address these issues and provide the research community with accessible high-quality research tools and models, MJFF developed a program to work with field leaders in tool/model development and key opinion leaders in the PD field to generate, characterize and distribute critical field-enabling research tools and preclinical models. The rationale and success for this strategy as it pertains to preclinical model development was first described in Baptista et al. [74].

One of biggest challenges for the PINK1-Parkin field has been the lack of sensitive antibodies that can detect endogenous levels of these proteins and the various downstream targets they impact. A complete understanding of the pathway in cellular and in vivo systems requires antibodies that are compatible with various assays such as immunoblotting, immunocytochemistry and immunohistochemistry and those that recognize various species of the protein so that findings can be easily extended from rodent models to human models. Unfortunately, the gold-standard antibody for detecting Parkin only works on some applications and does not bind to certain conformations of Parkin. Similarly, there are no sensitive antibodies that can detect different species of PINK1 or its activated state. The only approach to assess pathway activation successfully has been to measure phosphorylation of ubiquitin or Parkin and ubiquitination of downstream proteins driving mitophagy by mass spectrometry [46,75,76]. However, this platform is not routinely used by many labs and requires prior knowledge and expertise in the area along with the mass spectrometry equipment. The lack of sensitive, high-quality antibodies to measure different proteins and modifications in the PINK1-Parkin pathway presents a major bottleneck that the MJFF Preclinical Models and Tools Programs has been attempting to address.

The identification and validation of clear molecular players within the PINK1-Parkin mitophagy pathway has allowed the Foundation to prioritize the development, characterization, and distribution of 16 different tools/models for evaluating this pathway (Figure 1b, www.michaeljfox.org/toolscatalog). These targets have previously proved challenging for antibody development due to a variety of factors including the unstable nature of the PINK1 protein, the specific post-translational modifications that the antibodies must bind, and the low abundance/stoichiometry of some of these targets, among other reasons. By combining the expertise of researchers investigating the biology, biochemistry, and structure of these protein targets with the expertise of companies developing antibodies, MJFF hopes to develop high-quality, sustainable sources of these critical research tools to enable further investment and progress in the development of treatments and biomarkers for PD.

## 4. Conclusions

PINK1 and Parkin are compelling therapeutic targets and an area of growing interest for drug developers. The genetic link to disease, known crystal structure and a good understanding of the cellular function are encouraging researchers to develop therapies for this pathway. However, this pursuit is challenged by limited understanding of downstream modulators of neuroprotection or toxicity; lack of robust tools and tool compounds, and knowledge gaps around the role of this pathway in idiopathic PD for clinical trial design and patient selection. In order to tackle these roadblocks and ensure that scientific findings are robust and reproducible, The Michael J. Fox Foundation funds cutting-edge work, fosters collaborations, and contracts creation and distribution of critical research tools and resources. These efforts seek to build on a robust and growing PD therapeutic pipeline and to translate biological understanding of the PINK1-Parkin-mitochondrial pathway to identify new targets to one day enable the development of a cure for Parkinson’s disease.
